# Bedside ventilatory settings guided by respiratory mechanics in acute respiratory distress syndrome

**DOI:** 10.1186/s13613-025-01606-0

**Published:** 2025-11-29

**Authors:** Davide Chiumello, Silvia Coppola, Pedro Leme Silva, Giulia Lais, Patricia R. M. Rocco, Lise Piquilloud

**Affiliations:** 1 SC Anestesia e Rianimazione, ASST Santi Paolo e Carlo, Via Di Rudinì, Milan, Italy; 2 Department of Health Sciences, University of Milan, Milan, Italy; 3 Coordinated Research Center on Respiratory Failure, University of Milan, Milan, Italy; 4 Laboratory of Pulmonary Investigation, Carlos Chagas Filho Institute of Biophysics, Federal University of Rio de Janeiro, Rio de Janeiro, Brazil; 5 Adult Intensive Care Unit, University Hospital and University of Lausanne, Lausanne, Switzerland; 6 Department of Anesthesiology and Intensive Care Medicine, Catholic University of the Sacred Heart, and Anesthesia, Emergency and Intensive Care Medicine, Fondazione Policlinico Universitario A. Gemelli IRCCS, Rome, Italy

**Keywords:** Mechanical power, Driving pressure, Personalized mechanical ventilation, Subphenotypes

## Abstract

Ventilatory management of acute respiratory distress syndrome (ARDS) requires a careful balance between achieving adequate gas exchange and minimizing ventilator-induced lung injury (VILI). Recent advances in bedside monitoring of respiratory mechanics have created new opportunities to individualize mechanical ventilation by aligning ventilator settings with the patient’s dynamic pathophysiology. This review synthesizes current evidence on key respiratory mechanics parameters - such as driving pressure, respiratory system compliance, airway resistance, mechanical power - and examines how they can guide titration of tidal volume, positive end-expiratory pressure (PEEP), and respiratory rate. By integrating real-time assessments of respiratory mechanics, clinicians can reduce stress and strain, limit alveolar overdistension and collapse, and optimize oxygenation and ventilation. Moreover, practical strategies are discussed for implementing physiology-guided ventilation in the intensive care unit, with attention to patient-specific characteristics and the heterogeneity of ARDS subphenotypes. Respiratory mechanics-guided ventilation represents a pragmatic, individualized strategy that enhances lung protection, complements established protocols and may contribute to improve survival. Further experimental and clinical studies are required to validate these approaches and translate them into precision medicine for ARDS.

## Background

Acute respiratory distress syndrome (ARDS) is frequent in critically ill patients. When the Berlin definition is systematically applied, ARDS is identified in approximately 10% of all intensive care unit (ICU) admissions and in up to 23% of patients requiring invasive mechanical ventilation [1–3]. Severity is classified according to the PaO₂/FiO₂ ratio, which provides prognostic value, is used for clinical decisions such as prone positioning or extracorporeal support, and standardizes patient selection in research. In 2024, a new global definition was proposed as an update to the Berlin definition, expanding diagnostic criteria to include non-intubated patients supported with continuous positive airway pressure, non-invasive ventilation, or high-flow nasal oxygen (>40 L/min). It also allows the use of SpO₂/FiO₂ ≤ 315 (when SpO₂ ≤ 97%) as a surrogate for PaO₂/FiO₂, it incorporates lung ultrasound alongside chest radiography or CT, and it introduces flexibility for resource-limited settings [4]. These refinements aim to facilitate earlier diagnosis and broaden applicability across diverse healthcare systems. Nonetheless, both the Berlin and global definitions remain “static”, relying on a single oxygenation measurement despite substantial fluctuations that may occur within 24–48 h of mechanical ventilation [5, 6]. Recently, Bai et al.. identified three longitudinal oxygenation subgroups of ARDS (persistently low, gradually increasing and rapidly improving), which were more predictive of response to positive end-expiratory pressure (PEEP) and prognosis than the subgroups defined by a unique static PaO_2_/FiO_2_ ratio in the Berlin definition [7].

Since ARDS was first described in 1967 [8], the introduction of lung-protective ventilation - low tidal volumes (VT), limitation of alveolar pressures to reduce stress and strain and adequate PEEP to prevent collapse - has improved outcomes. Yet, mortality remains high, reaching 46% in severe cases (PaO₂/FiO₂ < 100 mmHg) [9]. Moreover, survivors often suffer long-term physical, cognitive, and psychological sequelae [10–12]. This highlights the potential limitations of the “one-size-fits-all” approach to treat ARDS patients. As an alternative, personalizing mechanical ventilation through real-time assessments of respiratory mechanics that integrate pathophysiological insights and identify patient-specific risks of overdistension or derecruitment may enable clinicians to optimize gas exchange while minimizing ventilator-induced lung injury (VILI). This could improve outcome. In addition, taking the ARDS phenotypes into account when setting the ventilator — particularly whether the patient has a hyper- or hypo-inflammatory phenotype — might also improve outcomes, even though these can change over time.

This review outlines the pathophysiological basis of ARDS, describes bedside assessments of respiratory mechanics, and explores how these parameters can guide ventilatory management, providing a physiology-based framework to advance personalized care. We also illustrate in this review how individualized settings based on respiratory mechanics go beyond a “one-size-fits-all” approach and really allows personalizing treatment.

## ARDS pathophysiology and the “Baby Lung” concept

ARDS represents a stereotyped response to diverse pulmonary or systemic insults, evolving through overlapping exudative and proliferative phases [13]. The initial injury may be direct (e.g., pneumonia, aspiration) or indirect (e.g., sepsis, trauma), triggering dysregulated inflammation, disruption of the alveolar-capillary barrier, epithelial and endothelial injury, and extracellular matrix degradation. These processes result in increased vascular permeability, impaired alveolar fluid clearance, and accumulation of protein-rich edema fluid within alveoli [14–16], yielding non-cardiogenic pulmonary edema and severe impairment of gas exchange.

A defining feature of ARDS is the spatial heterogeneity of lung involvement. Consolidated and non-aerated regions coexist with relatively preserved areas, giving rise to the concept of the “baby lung” - the portion of aerated, compliant lung available for ventilation [17, 18]. The inflammatory exudate increases lung weight and generates a gravitational gradient of superimposed pressure, compressing dependent regions and further reducing functional lung volume. Consequently, the “baby lung” is not a fixed structure but varies with body position and mechanical forces, and the ARDS lung has been described as a “mechanical sponge,” deformable under ventilation and gravity [19–21].

The anatomical and mechanical alterations observed in ARDS reduce respiratory system compliance (Crs), which is closely related to the volume of aerated lung, meaning to the “baby lung” [18, 21]. Notably, the intrinsic compliance of the aerated lung regions often remains near normal, indicating that the predominant limitation in ARDS is reduced functional lung size rather than increased tissue stiffness. This distinction is crucial for ventilatory management: protective strategies must be tailored to the limited and vulnerable “baby lung” in order to minimize VILI while maintaining adequate gas exchange.

### Core respiratory mechanics, including definitions

According to the equation of motion of the respiratory system, in passive patients, the pressure delivered by the ventilator must overcome both resistive forces and the elastic recoil of the lung and chest wall to achieve lung inflation (Table 1).

**Table 1:** Summary of respiratory mechanics parameters that can be assessed at the bedside, their physiological relevance and how they are calculated

Variables	How to calculate?	How to measure?	Why measure ?
*Equation of motion of the respiratory system (passive patient):* $$\begin{gathered} Paw{\text{ }} = {\text{ }}Pres{\text{ }} + {\text{ }}Pel{\text{ }} = {\text{ }} \hfill \\ \left( {Flow{\text{ }}x{\text{ }}R_{{AW}} } \right){\text{ }} + {\text{ }}\left( {Volume{\text{ }}x{\text{ }}E_{{RS}} } \right){\text{ }} + {\text{ }}PEEPtot \hfill \\ \end{gathered} $$			
*Respiratory system mechanics*			
Mean Airway Resistance (R_AW_)	$$ \begin{gathered} \,R_{{AW\,\left( {mean} \right)}} = \hfill \\ \frac{{P_{{peak}} - P_{{plat}} }}{{\,\dot{V}\,\left( {mean} \right)}} \hfill \\ \end{gathered} $$	End-inspiratory occlusion (i.e., in absence of flow) is needed to measure Pplat both during volume assist control and pressure assist control. During volume assist control with square inspiratory flow, inspiratory flow is constant and allows measuring mean inspiratory R_AW_.	It quantifies the impedance of the non-elastic component of the respiratory system to expand
Respiratory system elastance (E_RS_)	$\:Ers=\frac{{P}_{plat}-PEE{P}_{tot}}{{V}_{T}}$	End-inspiratory and end-expiratory occlusions (i.e., in absence of flow) during volume-controlled and pressure-controlled ventilation are needed to measure Pplat and PEEPtot.	It represents respiratory system stiffness; it is related to the amount of aerated lung. It is the reciprocal of C_RS_
Respiratory system compliance (C_RS_)	$\:Crs=\frac{{V}_{T}}{{P}_{plat}-PEE{P}_{tot}}$	End-inspiratory and end-expiratory occlusions (i.e., in absence of flow) during volume-controlled and pressure-controlled ventilation are needed to measure Pplat and PEEPtot.	Linearly related to the amount of aerated lung and thus to the baby lung size. It is the reciprocal of E_RS_
Driving pressure (ΔP)	$\:\varDelta\:P={P}_{plat}-PEE{P}_{tot}$	End-inspiratory and end-expiratory occlusions (i.e., in absence of flow) during volume-controlled and pressure-controlled ventilation are needed to measure Pplat and PEEPtot.	It quantifies the pressure used to expand the elastic component of the respiratory system, or in other words, reflects the distending force applied to the aerated lung. Targeting a maximal value allows adapting VT according the Crs in order to limit the distending pressure
Mechanical Power (MP)	$$\:M{P}_{VCV}=0.098\times\:RR\times\:{V}_{T}\times\:\left({P}_{peak}-\raisebox{1ex}{$\varDelta\:P$}\!\left/\:\!\raisebox{-1ex}{$2$}\right.\right)$$ $$\:M{P}_{PCV}=0.098\times\:RR\times\:{V}_{T}\times\:\left(PEEP+\varDelta\:P\right)$$	Same as ΔP for VCV.	It takes into account the frequency of the potential tidal damage imposed by the ventilator respiratory rate, and the effect of the increase of the resting volume provided by PEEP, computing the total energy delivered to the respiratory system.
Very simplified MP formula in ARDS (Costa simplified approach)	$\:CI=(4\times\:\varDelta\:P)+RR$	Same as ΔP.	Similar correlation with outcome in ARDS, compared to the more complicated formula
Recruitment-to-Inflation Ratio	$\:R/I\:=\frac{(Derecruited\:Volume/DeltaPEEP)}{Crs\:at\:low\:PEEP}$	Requires the measurement of expired volume during a high-to-low PEEP transition and of respiratory system compliance at low PEEP. Requires the absence of dynamic airtrapping and of airway closure at low PEEP.	It surrogates PV loop method to estimate lung recruitability: high values (i.e., towards 1) are associated with high lung recruitability
*Partitioned lung and chest wall mechanics*			
Transpulmonary pressure (P_L_)	$$\:{P}_{L}={(P}_{plat}-Pe{s}_{ei})$$ $$\:end-inspiratory{P}_{L}={P}_{plat}\times\:\raisebox{1ex}{${E}_{L}$}\!\left/\:\!\raisebox{-1ex}{${E}_{RS}$}\right.$$	Esophageal balloon is required to be properly positioned and calibrated. End-inspiratory and end-expiratory occlusions are also required.	Directly measured transpulmonary pressure at end-inspiration and end-expiration is influenced by the weight of the mediastinum. At end-inspiration, the directly measured PL better reflects the lung dependent regions Elastance-derived transpulmonary pressure at end-inspiration better reflects the non-dependent regions of the lung parenchyma
Chest wall compliance (C_CW_) Lung compliance (C_L_)	$\:{C}_{CW}=\frac{{V}_{T}}{Pe{s}_{ei}-Pe{s}_{ee}}$ $\:{C}_{L}=\frac{{V}_{T}}{{(P}_{plat}-Pe{s}_{ei})-(PEE{P}_{tot}-Pe{s}_{ee})}$	Esophageal balloon is required to be properly positioned and calibrated. End-inspiratory and end-expiratory occlusions are also required.	Separate contribution of the lung and chest wall mechanical properties

Paw: airway pressure, pres: resistive pressure, pel: elastic pressure, pplat: plateau pressure, peeptot: total positive end-expiratory pressure, crs: static respiratory system compliance, VT: tidal volume, cdyn: dynamic respiratory system compliance, ppeak: peak pressure, MP: mechanical power, RR: respiratory rate, ∆P: driving pressure, raw: airway resistance, PL: transpulmonary pressure, PPL pleural pressure, ecw: chest wall elastance, ers: respiratory system elastance, EL: lung elastance, pes: esophageal pressure. Ei: end-inspiration, ee: end-expiration

### Airway resistance

In mechanically ventilated patients, total airway resistance (Raw) reflects the combined resistance of the endotracheal tube, conducting airways, and lung tissue [22]. During volume-controlled ventilation with a constant (square) inspiratory flow, mean Raw can be estimated using the equation:

$$\:Raw\:\left(mean\right)=\frac{(Peak\:airway\:pressure-Plateau\:airway\:pressure)}{Inspiratory\:flow}$$

In clinical practice, Raw (mean) in intubated adults typically ranges from 5 to 10 cmH₂O/L/s. Increases in Raw - due to bronchospasm, airway secretions, or endotracheal tube obstruction - raise peak airway pressure (Ppeak) during volume-controlled ventilation, while plateau airway pressure (Pplat) remains unchanged, assuming the absence of intrinsic PEEP. During pressure-controlled ventilation, increases in Raw is associated with decreased inspiratory flow and lower VT.

### Compliance and elastance

Respiratory system elastance (Ers) quantifies the stiffness of the respiratory system and is defined as the change in transmural pressure per unit volume (Ers = ∆P/∆V). In practice, ∆P, in the absence of airway closure, equals plateau pressure (Pplat) measured during an end-inspiratory occlusion in the absence of flow -that corresponds to the alveolar pressure at the end of VT insufflation- minus total PEEP measured during an end-expiratory occlusion ((Ers = (Pplat – total PEEP) / VT). In ARDS, Ers is typically elevated due to alveolar collapse, “baby lung”, and interstitial oedema [19, 20].

**Fig. 1:**
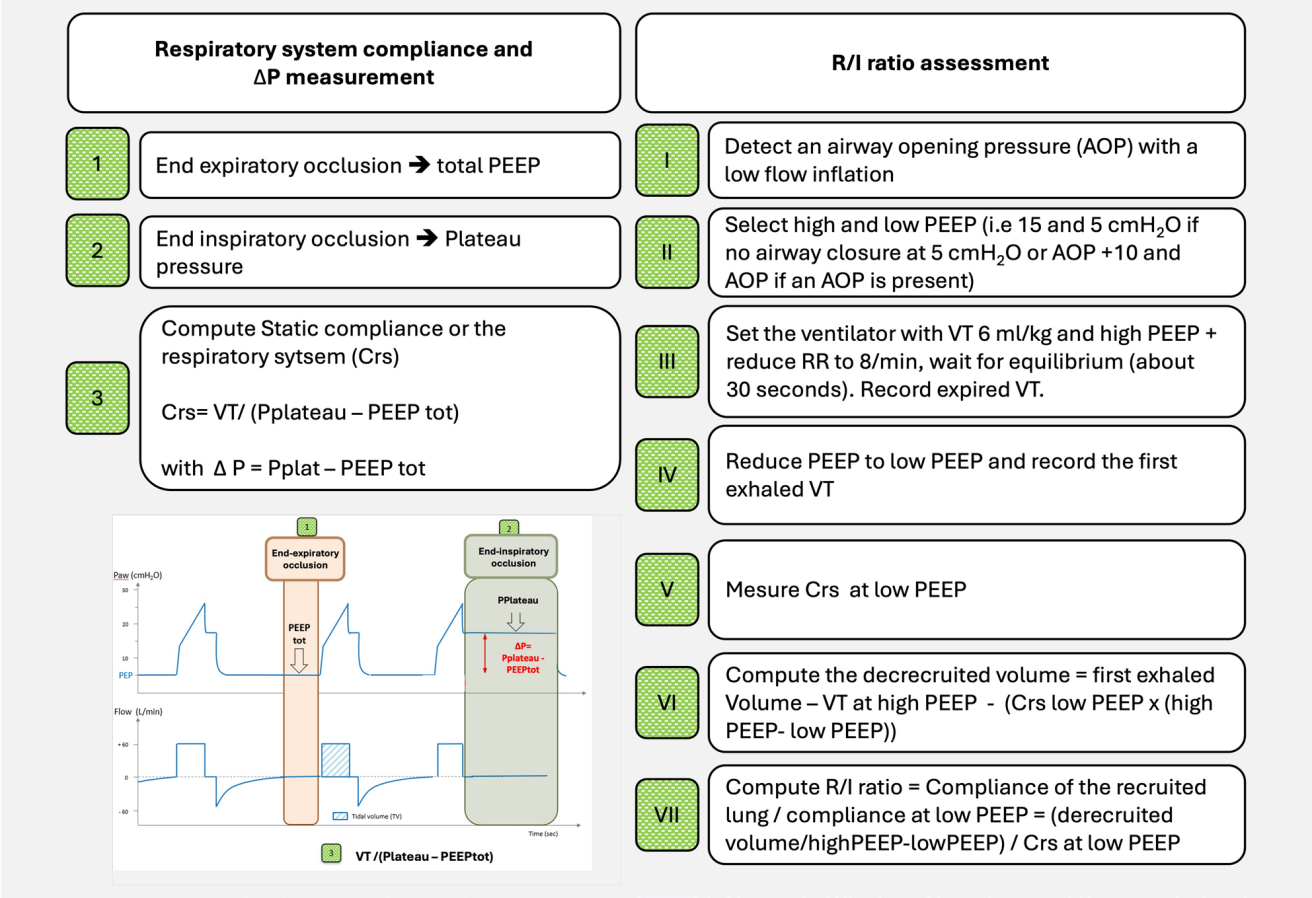
Illustration of the steps needed to assess driving pressure, respiratory system compliance (Crs) and recruitment over inflation ratio (R/I ratio). ∆P: driving pressure, PEEP: positive end-expiratory pressure, VT: tidal volume, Pplat: plateau pressure. AOP: airway opening pressure. RR: respiratory rate. To measure AOP, a low flow inflation (10 L/sec max) at low respiratory rate is needed. This can easily be done by adapting the ventilator settings in volume assist control. The AOP is present when a change in the slope of the airway pressure time curve is observed during the low flow inflation. The value of the AOP corresponds to the amount of pressure at the point of the change in the slope of the curve. If no AOP is present, the typical PEEP values to assess the R/I ratio are 5 and 15 cmH_2_O. In presence of an AOP, the low PEEP value to assess the R/I ratio should be equal to the AOP. The high PEEP value is thus ideally AOP + 10 cmH_2_O except if the resultant PEEP. is really too high for a given patient. In this specific situation, select a high PEEP lower that AOP + 10 according to the patient tolerance. The steps required to assess the R/I ratio are detailed in the right panel. Reference for R/I ratio measurement. Chen et al. Potential for Lung Recruitment Estimated by the Recruitment-ti-Inflation ratio in Acute Respiratory Distress Syndrome. A clinical trial. Am J Respir Crit Care Med. 2020 15:201 [2]:178–187. Online calculator: https://rtmaven.com/ri-ratio

Respiratory system compliance (Crs = ∆V/∆P)the reciprocal of Ers, reflects distensibility [23, 24]. Under static conditions (i.e., zero flow), Crs is measured using end-inspiratory and end-expiratory occlusion maneuvers [23]:

$$\:Crs=\frac{VT}{\left(Pplat-PEEPtot\right)}$$

where: VT is tidal volume; Pplat is plateau airway pressure measured at end-inspiration during an end-inspiratory occlusion, and PEEPtot is the total end-expiratory airway pressure (including both extrinsic and intrinsic PEEP). Measurement steps are illustrated in Fig. 1 (left panel).

In healthy, spontaneously breathing individuals, Crs typically ranges from 100 to 200 mL/cmH₂O. In intubated patients, Crs decreases to 50–80 mL/cmH₂O mainly due to reductions in functional residual capacity, but also to dynamic external pressures, such as elevated intra-abdominal pressure, pleural effusions, or chest wall edema. To note, constant loads like obesity primarily shift the pressure–volume curve without necessarily reducing compliance [7].

An approximate prediction of normal Crs in intubated patients based on vital capacity (VC) is 1.6% of VC [25], where VC estimated using sex-specific formulas:

For men:$$ \begin{gathered} \,VC\left( {mL} \right) = \hfill \\ \left( {5.76 \times \,height\,\left[ m \right] - 0.026 \times \,age\,\left[ {years} \right] - 4.34} \right) \times \,1000 \hfill \\ \end{gathered} $$

For women:

$$ \begin{gathered} \,VC\,\left( {mL} \right) = \hfill \\ \left( {4.43 \times \,height\,\left[ m \right] - 0.026 \times \,age\,\left[ {years} \right] - 2.89} \right) \times \,1000 \hfill \\ \end{gathered} $$

Driving pressure (DP) is defined as the difference between Pplat and PEEPtot [26].

$$\:\varDelta\:P=Pplat-PEEPtot$$

Based on the Crs equation, $\:\varDelta\:P\:$can also be written as:

$$\:\varDelta\:P=Pplat-PEEPtot=\frac{VT}{Crs}$$

DP reflects the distending force applied to the “baby lung” and is a key determinant of VILI [26, 27]. A reduction in ΔP following PEEP increase may indicate successful lung recruitment. In addition, targeting ΔP ≤ 15 cmH₂O—ideally lower—has been associated with improved survival in ARDS [28–30]. Notably, higher ΔP increases lung stress even when tidal volume (VT) and PEEP are within conventional limits [31]. Current guidelines recommend VT of 4–8 mL/kg predicted body weight in ARDS [32, 33]. However, heterogeneity is the norm in ARDS, which clearly challenges the idea of using a similar VT for all the patients. Heterogeneity encopasses not only the size of the “baby lung” but also the influence of patient sex and ethnicity on normal lung volume. Ongoing research aims to determine whether ΔP can serve not only as a physiologically relevant prognostic marker but also as a mean of guiding individualized VT adjustments according to the “baby lung” and the individual patient characteristics in order to optimize ventilation, limit the lung-distending pressure and reduce mortality.

### Partitioning lung and chest wall mechanics

To separate lung and chest wall contributions to total respiratory system elastance and compliance, esophageal pressure (Pes) can be measured using a catheter with an esophageal balloon positioned in the distal esophagus [34–37]. This allows the direct calculation of transpulmonary pressure (PL) at end-inspiration (ei):

$$\:PL\:ei=Pplat-Pes\:ei$$

and at end-expiration (ee):$\:PL\:ee=PEEPtot-Pe{s}_{}ee$

Lung (CL) and chest wall (Ccw) compliance can then be calculated as:

$$\:CL=\frac{VT}{\left(Pplat-{Pes\:ei}_{\:}\right)-(PEEPtot-Pes\:ee)}$$

$$\:Ccw=\frac{VT}{Pes\:ei\:i-Pes\:ee}$$

where Pes_ee is the esophageal (i.e., surrogate of pleural) pressure at end-expiration.

Similarly, ERS can be partitioned in l chest wall elastance (ECW) and lung elastance (EL)

$$\:ECW=\frac{Pes\:ei\:i-Pes\:ee}{VT}$$

$$\:EL=\frac{\left(Pplat-{Pes\:ei}_{\:}\right)-(PEEPtot-P\:es\:ee)}{VT}$$

$$\:or\:$$

$$\:EL=ERS-ECW$$.

Partitioned values are particularly relevant in conditions affecting chest wall mechanics, such as intra-abdominal hypertension or chest wall edema, where airway pressures may not accurately reflect true lung-distending pressures.

It should be noted that direct measurement of PL at end-inspiration as described above does not account for vertical pleural pressure gradients in the supine patient, with higher pressures in dorsal (dependent) and lower in ventral (non-dependent) lung regions [38] due to superimposed pressure. In fact, the direct method reflects PL in the region surrounding the esophagus, which corresponds to the dependent lung region [39]. Another approach, the elastance-derived method, estimates PL without relying on absolute Pes values:

$$\:PL,ei=Pplat\times\:\frac{{E}_{L}}{{E}_{RS}}$$

This approach better represents PL in non-dependent lung regions [39], which are more prone to overdistension. However, it assumes pleural pressure is zero at functional residual capacity, which is not always accurate. PL can be used to set PEEP as described elsewhere [35, 36].

### Mechanical power

Mechanical power (MP) quantifies the energy transferred from the ventilator to the respiratory system per unit time, incorporating both static (elastic) and dynamic (resistive and inertial) components of ventilation [40, 41]. MP integrates VT, airflow, respiratory rate (RR), PEEP, and respiratory system mechanics, with its calculation dependent on the mode of mechanical ventilation.

Because complete calculation at the bedside is often impractical, simplified equations have been proposed for clinical use [42] :

Volume-controlled ventilation:

$$\:MP=0.098\times\:RR\times\:VT\times\:\left[\right(PPeak-0.5\times\:\left(\varDelta\:P\right)]$$

Pressure-controlled ventilation:

$$\:MP=0.098\times\:RR\times\:VT\times\:(PEEP+\varDelta\:P)$$

Recent studies have shown that a simplified surrogate for MP proposed by Costa et al. and calculated as (4 × ΔP) + RR, provides predictive performance comparable to full mechanical power calculations in ARDS patients. Using this approach, ventilatory strategies can be individualized: patients with low CRS may have improved outcome by lowering ΔP through VT adjustment as this will have a major impact on reducing MP, whereas patients with higher CRS may not derive the same benefit [43].

To note, a universally accepted MP threshold for VILI remains undefined. Observational studies report varying cut-offs: MP >12 J/min after 24 h in ARDS increased 90-day mortality [44], MP >17 J/min was associated with higher hospital length-of-stay and mortality [45], and MP >22 J/min correlated with fewer ventilator-free days and higher 28-day and 3-year mortality [46]. Therefore, no single MP limit can yet be recommended clinically [47, 48].

### Respiratory mechanics as a target for ventilator setting personalization

Ers reflects the mechanical load on the lungs and is a major determinant of stress and strain. The mortality benefit of low-VT strategies is most pronounced in patients with high Ers, where reduction of VT limits injurious overdistension [49]. In those with lower Ers, excessively low VT may not confer added protection and could impair gas exchange. This principle is reinforced by secondary analyses of randomized trials, which demonstrated that the benefit of neuromuscular blockade was confined to patients with high Ers. Notably, this interaction was absent for other physiologic or biomarker variables, suggesting that Ers is a particularly robust and actionable parameter to guide ventilation and sedation strategies [50].

### Practical applications of the respiratory mechanics concepts

#### Using Crs and DP to assess the “baby lung” and personalize VT

Gattinoni and colleagues demonstrated that Crs reflects the functional size of the “baby lung” in ARDS [18, 21]. Lower Crs indicates a smaller “baby lung”, which is more vulnerable to overdistension and VILI. Because ΔP is defined as VT/Crs, limiting ΔP at a fixed PEEP level inherently adjusts VT to the “baby lung”. This physiology-based approach minimizes stress and strain and could help preventing mechanical injury.

#### Using respiratory mechanics concepts to assess lung recruitability and optimize PEEP setting

Optimizing PEEP requires evaluating lung recruitability. While higher PEEP can reopen collapsed alveoli and expand the “baby lung,” it may also promote overdistension, increase dead space, and impair hemodynamics, particularly in patients with low recruitability [51, 52]. Notably, in the seminal work of Gattinoni et al. on lung recruitability in ARDS, recruitable patients, defined as those who gained at least 9% of aerated lung volume at a higher pressure, represented only around half of the patients [52].

Although CT imaging across PEEP levels remains the gold standard to assess recruitability, it is rarely feasible at the bedside. An alternative approach is the analysis of the pressure–volume curves between two PEEP levels combined with respiratory mechanics measurements, which allows quantifying changes in lung volume. This method partitions the observed volume change (ΔVtot) into:

1. Predicted DEELV-PEEP due to PEEP increase only (volume increase expected solely from the applied PEEP increment, without recruitment).

$$\:Predicted\:\varDelta\:EELV-PEEP=\varDelta\:PEEP\times\:Crs\:at\:low\:PEEP$$

2) DVrecruit (volume increase due to recruitment)

$$ \begin{gathered} \,\Delta \,{\text{Vrecruit}} = \hfill \\ \,\Delta \,{\text{Vtot}}\, - {\text{predicted}}\Delta \,{\text{EELVPEEP}} \hfill \\ \end{gathered} $$.

where $\:\varDelta\:\text{V}\text{t}\text{o}\text{t}$ is the total change in volume between the two levels of PEEP.

Although physiologically informative, this technique is rarely used in routine practice because of its technical complexity [53].

Based on the same principles, the Recruitment-to-Inflation Ratio (R/I) has been proposed by Chen et al. as a simpler bedside index of recruitability. It requires, in the absence of dynamic airtrapping (which requires to decrease respiratory rate to be assessed), only a rapid reduction in PEEP within a clinically appropriate range (e.g., from 15 to 5 cmH₂O if no airway closure is present at 5 cmH₂O, or from airway opening pressure (AOP) + 10 to AOP if an AOP is present) with measurement of the first exhaled volume after the decrease in PEEP level and some calculations. The first exhaled volume after the decrease in PEEP equals the sum of VT, predicted DEELV-PEEP (computed as previously described as DPEEP x Crs at low PEEP) and derecruited volume (Vrecruit). The derecruited volume—reflecting the amount previously recruited by higher PEEP—is thus calculated as:

Vrecruit = first exhaled volume after the decrease in PEEP minus VT minus predicted DEELV-PEEP.

**Fig. 2:**
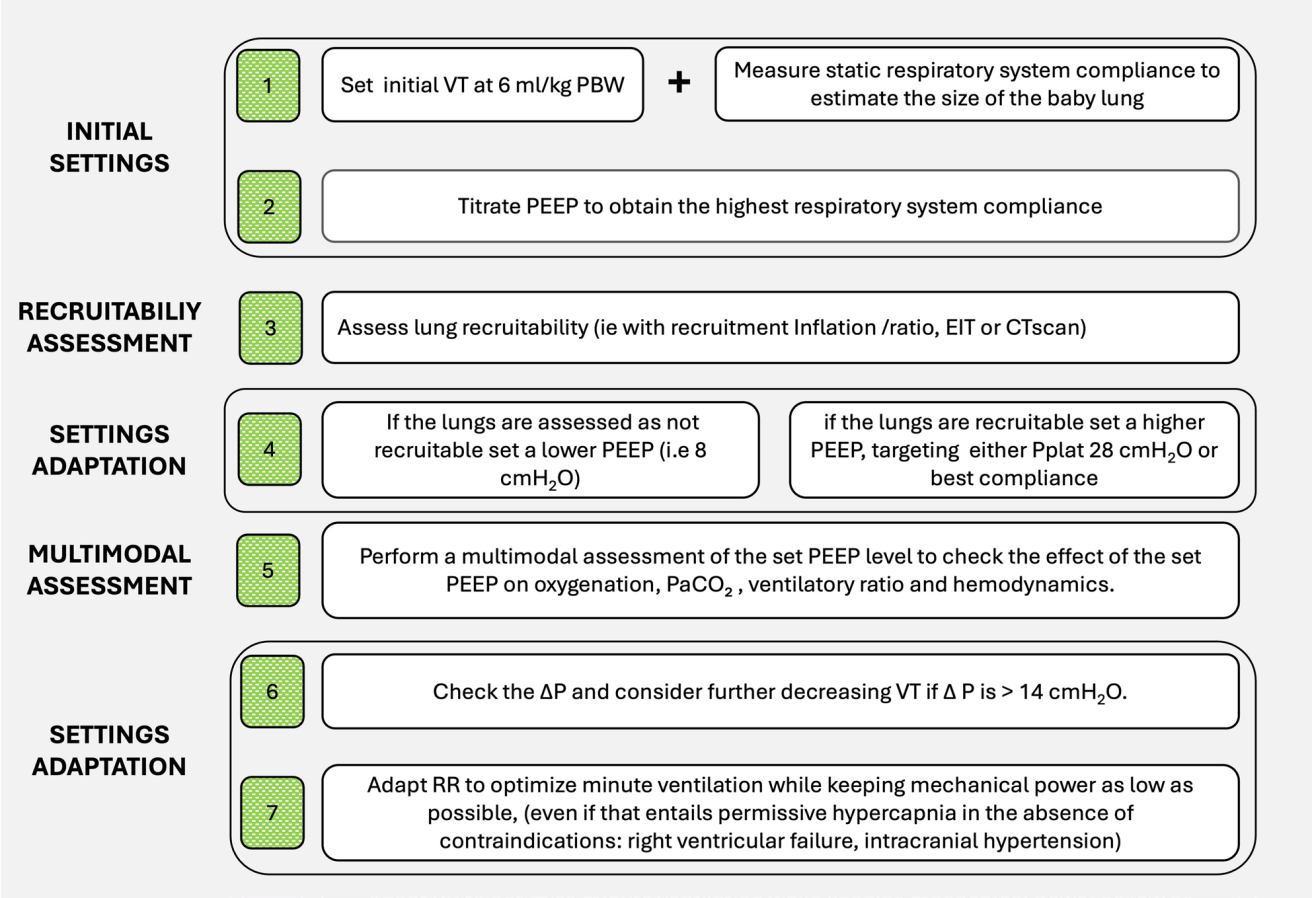
Illustration of the stepwise approach to optimize the ventilator settings based on respiratory mechanics. VT: tidal volume. PBW: predicted body weight, PEEP: positive end-expiratory pressure, PV curves: pressure volume curves, ∆P: driving pressure, RR: respiratory rate, CT computed tomography

The R/I ratio is then calculated as the compliance of the (de)recruited lung (that equals $\:{\Delta\:}\text{V}\text{r}\text{e}\text{c}\text{r}\text{u}\text{i}\text{t}$/deltaPEEP) divided by the Crs at low PEEP (Fig. 2).

$$\:R/I\:=\frac{(Derecruited\:Volume/deltaPEEP)}{Crs\:at\:low\:PEEP}$$

An R/I ratio < 0.3 indicates low recruitability and suggests limited benefit from high PEEP, whereas a ratio >0.6 suggests substantial recruitability. Intermediate values (0.3–0.6) should be interpreted cautiously, in the context of other clinical and physiological data such as plateau pressure, ΔP, and hemodynamic status. Importantly, the R/I ratio should be viewed as a continuous variable reflecting the trade-off between recruitment and hyperinflation, and its clinical validation remains limited [54–56]. In addition, performing the R/I ratio might be challenging or even risky in the most hypoxemic patients.

#### Using respiratory mechanics concepts to set the ventilator during volume- and pressure-controlled modes

A physiology-guided, stepwise approach can be proposed to optimize ventilatory settings in deeply sedated or paralyzed ARDS patients receiving volume assist-control ventilation. The overarching goal is to achieve adequate gas exchange while minimizing VILI by tailoring PEEP and VT to individual lung mechanics. This algorithm (Fig. 2) is based on strong physiological rationale but has not yet been validated in randomized prospective trials.

##### Step 1: estimate “baby lung” and assess VT tolerability

Initiate ventilation with a VT of 6 mL/kg predicted body weight (PBW) and measure Crs at a baseline PEEP of 5 cmH₂O to estimate the “baby lung”. If available, Pes monitoring may help with partition lung and chest wall compliances.

- **Normal or near-normal Crs (50–100 mL/cmH₂O)**: Suggests that much of the lung is already aerated. In this setting, assess the risk of overdistension and reduce VT if necessary to maintain ΔP < 14 cmH₂O.
- **Low Crs**: Indicates a smaller “baby lung” and the need to evaluate potential recruitment with PEEP adjustments.

##### Step 2: if Crs is low, perform initial PEEP Titration to maximize Crs

Incrementally increase PEEP to identify the lowest level at which Crs is maximized. This might help finding an initial balance between optimal recruitment and not too much overdistension. However, it only provides an individualized starting point for further titration. Indeed, in specific situations, very low PEEP has been associated with the best compliance likely due to intra-tidal recruitment [57]. Oppositely, in the ART trial, the opened lung strategy that focused on recruitment maneuvers and subsequent Crs-maximization was associated with increased mortality [58].

##### Step 3: assess lung recruitability

Recruitability may be assessed using:

- CT at different PEEP levels (gold standard).
- Pressure–volume curves.
- Recruitment-to-Inflation (R/I) ratio.
- Electrical impedance tomography (EIT), when available [51].

These tools distinguish highly recruitable patients likely to benefit from higher PEEP from those at risk of overdistension.

##### **Step 4: adjust PEEP based on recruitability**

- **Low recruitability**: Favor a lower PEEP strategy (5–8 cmH₂O) to limit overdistension and hemodynamic compromise.
- **High recruitability**: Consider higher PEEP, guided by either a target Pplat ≤ 28 cmH₂O [59] or the PEEP associated with maximal Crs while limiting ΔP ≤ 14 cmH₂O.

##### Step 5: reassess gas exchange and hemodynamics

Evaluate the effects of the chosen PEEP on:

1. gas exchange: PaO₂, PaCO₂, and ventilatory ratio (as a surrogate for dead space),
2. hemodynamics: Arterial pressure, echocardiography, or invasive cardiac output monitoring.

Optimal PEEP should maximize oxygen delivery, not merely PaO₂ [60].

##### Step 6: target driving pressure

If ΔP exceeds 14 cmH₂O, reduce VT if feasible, provided it does not result in severe hypercapnia or acidosis.

##### Step 7: calculate and minimize mechanical power

Adjust RR to achieve acceptable minute ventilation while keeping MP—the energy delivered to the lung per unit time—as low as possible. Permissive hypercapnia is generally tolerated, but caution is warranted in patients with right ventricular dysfunction (hypercapnia-induced pulmonary vasoconstriction) [61] or acute brain injury (risk of raised intracranial pressure) [62].

The same physiological principles apply to pressure -control ventilation but it is essential to measure Pplat during an end-inspiratory occlusion as inspiratory pressure equals plateau pressure only if flow returns to zero before end-inspiration. Measuring Pplat permits calculation of ΔP and Crs, as in volume assist-control.

#### Using respiratory mechanics concepts to set the ventilator during assisted breathing

The transition from controlled to assisted ventilation requires strict attention to limiting stress and strain to avoid patient self-inflicted lung injury (P-SILI) [63]. Driving pressure and respiratory system compliance can still be measured in spontaneously breathing patients. This is done through an end-inspiratory occlusion during a patient-triggered breath, which provides Pplat and, therefore, the total distending pressure of the respiratory system—reflecting both inspiratory muscle effort and the positive pressure delivered by the ventilator [64]. Reliable Pplat measurements are often feasible during assisted breathing [64], and high early Pplat has been associated with poor outcomes [65, 66]. Dynamic transpulmonary pressure (∆PLdyn) offers further assessment of the lung distending pressure. It represents the combined fall in pleural pressure generated by inspiratory muscles and the positive pressure above PEEP delivered by the ventilator [67]. The gold standard for pleural pressure measurement is an esophageal balloon, but when unavailable, the drop in airway pressure during an end-expiratory occlusion in pressure support mode (Pocc) is a practical alternative [67, 68]. Multiplying Pocc by 0.66 provides a surrogate of the esophageal pressure swing, allowing calculation of ∆PLdyn as (∆Peso during occlusion + pressure support above PEEP) [68]. This is a dynamic parameter influenced by airway resistance; a safety threshold of 22 cmH₂O has been suggested, although interpretation must consider conditions that increase resistance (e.g., COPD, small endotracheal tube, secretions) [67]. These physiological assessments inform ventilator optimization. If Pplat and/or ∆PLdyn are elevated but inspiratory muscle pressure (Pmus, estimated as Pocc × 0.75) is low (5–10 cmH₂O), over-assistance is likely, and pressure support should be reduced. Conversely, if Pmus is high (>10 cmH₂O), under-assistance may be suspected, and a gradual increase in pressure support is warranted. If this reduces Pmus while maintaining safe Pplat and ∆PLdyn, settings are appropriate. Persistently high Pmus despite increased support may require additional strategies, including PEEP titration or sedation (propofol, benzodiazepines). If these fail, returning to controlled ventilation should be considered, as excessive drive and effort during transition have been linked to relapse of acute hypoxemic respiratory failure. To note, setting the ventilator based on this monitoring st during assisted breathing has not been validated in prospective studies.

## Other personalization tools: subphenotypes, imaging and age

ARDS is a pathophysiologically heterogeneous syndrome, encompassing diverse lung morphologies, respiratory mechanics, and systemic responses. This heterogeneity profoundly influences ventilatory management and outcomes. For example, clinically based phenotypes such as the severity of hypoxemia are used in practice to guide treatments for specific subgroups of patients known to benefit from them. Prone positioning, for instance, is recommended when PaO_2_/FiO_2_ is lower than 150 mmHg after ventilator settings have been optimized. Another example is the need for caution when using non invasive ventilation as first line respiratory support in patients with severe hypoxemia, because in these patients, this strategy has been associated with increased hospital mortality. Personalized ventilation seeks to align treatment with individual patient profiles by integrating subphenotype classification, lung morphology, respiratory mechanics, age, and comorbid conditionsm [69–72].

### ARDS subphenotypes: hyperinflammatory vs. hypoinflammatory

Accumulating evidence supports biologically distinct ARDS subphenotypes that respond differently to ventilatory interventions [57]. The hyperinflammatory phenotype is characterized by elevated cytokines, vascular leak, and multiorgan dysfunction, often with low compliance and severe hypoxemia. These patients may benefit from higher PEEP to mitigate atelectrauma and enhance alveolar recruitment [60]. Conversely, the hypoinflammatory phenotype generally exhibits preserved compliance, less severe hypoxemia, and stable systemic physiology; in this setting, aggressive PEEP may promote overdistension and impair hemodynamics [60].

Most subphenotyping studies rely on cross-sectional data, limiting their ability to capture ARDS dynamics. A recent three-class framework integrated physiology, CT findings, and clinical outcomes has been proposed [61]:

- **Class 1**: Minimal organ dysfunction and near-normal laboratory values.
- **Class 2**: Severe respiratory failure, with low PaO₂/FiO₂, high ΔP, MP, and ventilatory ratio.
- **Class 3**: Predominantly extrapulmonary involvement with elevated lactate, renal dysfunction, and vasopressor needs.

Importantly, many patients transitioned between phenotypes during the first four days of ventilation, underscoring the need for adaptive monitoring of physiology and respiratory mechanics.

### Imaging-based subphenotyping

Radiological assessment provides an additional dimension for personalization. In focal ARDS, injury is confined to dependent lung regions while nondependent areas remain aerated. These patients are prone to overdistension with high PEEP and often benefit from lower VT, moderate PEEP, and avoidance of recruitment maneuvers [62–64, 73],. In diffuse ARDS, characterized by widespread alveolar collapse and inflammation, higher PEEP, recruitment maneuvers, and prone positioning are generally more effective in maintaining alveolar stability and optimizing ventilation–perfusion matching. Incorporating CT or EIT into bedside decision-making is therefore critical to reduce iatrogenic injury [65].

### Age: a crucial modifier of ventilatory strategy

Elderly patients (≥ 80 years)—a growing demographic in the ICU—present unique challenges due to age-related changes in lung mechanics and increased vulnerability to VILI [74, 75]. A large, pooled analysis from seven ARDS Network and PETAL trials found that the relationship between ΔP and mortality is significantly amplified in this population [76]. While ΔP thresholds of 14 cmH₂O are generally accepted, a lower target of ≤ 11 cmH₂O may be more appropriate in very elderly patients to avoid barotrauma and enhance survival.

These data advocates for age-adjusted ventilatory strategies, recognizing that frail patients may require more conservative targets to mitigate harm.

## Emerging technologies: AI and omics

Artificial intelligence (AI) is revolutionizing the diagnosis, management, and prognosis of ARDS by utilizing large datasets and advanced computational models. AI techniques, including machine learning (ML) [77, 78], deep learning (DL) [79] and natural language processing [80], enable the identification of ARDS subphenotypes, improve outcomes prediction, and might help optimizing mechanical ventilation.

AI-based algorithms can analyze heterogeneous data sources—including electronic health records, imaging modalities (e.g., chest X-rays and CT scans) [81, 82], and real-time physiological signals—to enhance early ARDS recognition, often outperforming conventional clinical assessments [83]. DL models, in particular, can augment diagnostic precision and reduce delays in initiating evidence-based therapy [79].

Prognostic models integrating clinical variables, respiratory mechanics, and biomarkers have been developed to estimate mortality risk, likelihood of ventilator dependence, and therapeutic responsiveness [84]. Moreover, AI systems can continuously process ventilator waveforms to assist with the dynamic adjustment of VT, PEEP, and ΔP, while detecting patient–ventilator dyssynchrony [85] and predicting weaning success [86]. Reinforcement learning techniques might further contribute to the optimization of lung-protective ventilation by minimizing VILI.

Beyond ventilatory management, AI enhances image interpretation, enabling automated quantification of lung abnormalities and severity classification. Omics-integrated AI platforms (e.g., proteomics and transcriptomics) are also advancing the discovery of novel biomarkers to support precision medicine in ARDS [87]. Importantly, decision-support systems powered by AI can synthesize multimodal data to assist clinicians with high-stakes decisions, such as timing of weaning, sedation modulation, and proning [86, 88].

Despite its promise, the clinical implementation of AI in ARDS care remains limited by issues of data heterogeneity, model interpretability, and workflow integration. To realize its full potential, future research must prioritize external validation, regulatory approval, and strategies that foster human–AI collaboration to ensure real-world applicability and safety.

### Summary and future directions

Optimizing ventilatory management in ARDS necessitates a sophisticated, real-time integration of respiratory physiology at the bedside. Core parameters—including Crs, Ers, ΔP and lung recruitability—provide critical insights that help individualizing ventilator settings. These physiologic markers enable clinicians to estimate the “baby lung”, evaluate the potential for recruitment, and titrate ventilation by selecting the appropriate VT, PEEP and RR to minimize ΔP and MP, thereby mitigating VILI while preserving gas exchange.

Advanced tools such as the recruitment-to-inflation ratio (R/I) and emerging AI-driven predictive models hold promise for further enhancing bedside assessment and personalizing ventilatory strategies. By moving beyond standardized protocols and embracing a physiology-guided, patient-specific approach, clinicians can align mechanical ventilation with the dynamic and heterogeneous nature of ARDS. This paradigm not only embodies the principles of lung protection but also holds the potential to improve both short- and long-term patient outcomes.

## Data Availability

Not applicable.

## Abbreviations

AI: Artificial intelligence
ARDS: Acute Respiratory Distress Syndrome
Ccw: Chest wall compliance
Cdyn: Dynamic compliance of the respiratory system
CL: Lung compliance
Crs: Respiratory system compliance
CT: Computed tomography
DL: Deep learning
DP: Driving pressure
DVrecruit: change in volume attributable to recruitment
EIT: Electrical impedance tomography
Ers: Elastance of the respiratory system
ICU: Intensive Care Unit
ML: Machine learning
MP: Mechanical power
PBW: predicted body weight
Pes: Esophageal pressure
Pes_insp: Esophageal pressure at end-inspiration
Pes_exp: Esophageal pressure at end-expiration
Ppeak: Peak inspiratory pressure
Pplat: Plateau pressure
PEEP: positive end-expiratory pressure
PEEPtot: Total PEEP
Raw: Total airway resistance
R/I ratio: Recruitment-to-Inflation ratio
RR: Respiratory rate
VILI: Ventilator-Induced Lung Injury
VC: Vital capacity
VT: Tidal volume

## References

[1] Ferguson ND, Fan E, Camporota L, Antonelli M, Anzueto A, Beale R, et al. The Berlin definition of ARDS: an expanded rationale, justification, and supplementary material. Intensive Care Med. 2012;38(10):1573–82. 22926653 10.1007/s00134-012-2682-1

[2] Bellani G, Laffey JG, Pham T, Fan E, Brochard L, Esteban A, et al. Epidemiology, patterns of care, and mortality for patients with acute respiratory distress syndrome in intensive care units in 50 countries. JAMA. 2016;315(8):788–800. 26903337 10.1001/jama.2016.0291

[3] Bellani G, Laffey JG, Pham T, Fan E. The LUNG SAFE study: a presentation of the prevalence of ARDS according to the Berlin Definition! Crit Care (London England). 2016;20:268. 10.1186/s13054-016-1443-x PMC5016866 27608629

[4] Matthay MA, Arabi Y, Arroliga AC, Bernard G, Bersten AD, Brochard LJ, et al. A new global definition of acute respiratory distress syndrome. Am J Respir Crit Care Med. 2024;209(1):37–47. 37487152 10.1164/rccm.202303-0558WS PMC10870872

[5] Nasa P, Bos LD, Estenssoro E, van Haren FMP, Neto AS, Rocco PRM, et al. Defining and subphenotyping ARDS: insights from an international Delphi expert panel. Lancet Respir Med. 2025;13(7):638–50. 40315883 10.1016/S2213-2600(25)00115-8

[6] Pozzi T, Fratti I, Tomarchio E, Bruno G, Catozzi G, Monte A, et al. Early time-course of respiratory mechanics, mechanical power and gas exchange in ARDS patients. J Crit Care. 2024;79:154444. 37862955 10.1016/j.jcrc.2023.154444

[7] Bai Y, Chen S, Yang H, Huang X, Xia J, Zhan Q. Dynamic oxygenation subgroup bringing new insights in ARDS: more predictive of outcomes and response to PEEP than static PaO(2)/FiO(2). Thorax. 2025;80(9):594–603. 40393717 10.1136/thorax-2024-222360

[8] Ashbaugh DG, Bigelow DB, Petty TL, Levine BE. Acute respiratory distress in adults. Lancet (London England). 1967;2(7511):319–23. 4143721 10.1016/s0140-6736(67)90168-7

[9] Pham T, Pesenti A, Bellani G, Rubenfeld G, Fan E, Bugedo G et al. Outcome of acute hypoxaemic respiratory failure: insights from the LUNG SAFE study. Eur Respir J. 2021;57(6). 10.1183/13993003.03317-2020 33334944

[10] Herridge MS, Tansey CM, Matté A, Tomlinson G, Diaz-Granados N, Cooper A, et al. Functional disability 5 years after acute respiratory distress syndrome. N Engl J Med. 2011;364(14):1293–304. 21470008 10.1056/NEJMoa1011802

[11] Herridge MS, Moss M, Hough CL, Hopkins RO, Rice TW, Bienvenu OJ, et al. Recovery and outcomes after the acute respiratory distress syndrome (ARDS) in patients and their family caregivers. Intensive Care Med. 2016;42(5):725–38. 27025938 10.1007/s00134-016-4321-8

[12] Rothenhäusler HB, Ehrentraut S, Stoll C, Schelling G, Kapfhammer HP. The relationship between cognitive performance and employment and health status in long-term survivors of the acute respiratory distress syndrome: results of an exploratory study. Gen Hosp Psychiatry. 2001;23(2):90–6. 11313077 10.1016/s0163-8343(01)00123-2

[13] Rocco PR, Negri EM, Kurtz PM, Vasconcellos FP, Silva GH, Capelozzi VL, et al. Lung tissue mechanics and extracellular matrix remodeling in acute lung injury. Am J Respir Crit Care Med. 2001;164(6):1067–71. 11587998 10.1164/ajrccm.164.6.2007062

[14] Ball L, Silva PL, Giacobbe DR, Bassetti M, Zubieta-Calleja GR, Rocco PRM, et al. Understanding the pathophysiology of typical acute respiratory distress syndrome and severe COVID-19. Expert Rev Respir Med. 2022;16(4):437–46. 35341424 10.1080/17476348.2022.2057300 PMC9115784

[15] Rocco PR, Pelosi P. Pulmonary and extrapulmonary acute respiratory distress syndrome: myth or reality? Curr Opin Crit Care. 2008;14(1):50–5. 18195626 10.1097/MCC.0b013e3282f2405b

[16] Al-Husinat L, Azzam S, Al Sharie S, Al Sharie AH, Battaglini D, Robba C, et al. Effects of mechanical ventilation on the interstitial extracellular matrix in healthy lungs and lungs affected by acute respiratory distress syndrome: a narrative review. Crit Care (London England). 2024;28(1):165. 10.1186/s13054-024-04942-y PMC11094887 38750543

[17] Gattinoni L, Marini JJ, Pesenti A, Quintel M, Mancebo J, Brochard L. The baby lung became an adult. Intensive Care Med. 2016;42(5):663–73. 26781952 10.1007/s00134-015-4200-8

[18] Gattinoni L, Pesenti A. The concept of baby lung. Intensive Care Med. 2005;31(6):776–84. 15812622 10.1007/s00134-005-2627-z

[19] Cortes-Puentes GA, Gard KE, Adams AB, Dries DJ, Quintel M, Oeckler RA, et al. Positional effects on the distributions of ventilation and end-expiratory gas volume in the asymmetric chest-a quantitative lung computed tomographic analysis. Intensive Care Med Experimental. 2018;6(1):9. 10.1186/s40635-018-0175-4 PMC5891440 29633056

[20] Thompson BT, Chambers RC, Liu KD. Acute respiratory distress syndrome. N Engl J Med. 2017;377(6):562–72. 28792873 10.1056/NEJMra1608077

[21] Gattinoni L, Caironi P, Pelosi P, Goodman LR. What has computed tomography taught Us about the acute respiratory distress syndrome? Am J Respir Crit Care Med. 2001;164(9):1701–11. 11719313 10.1164/ajrccm.164.9.2103121

[22] Pesenti A, Pelosi P, Rossi N, Virtuani A, Brazzi L, Rossi A. The effects of positive end-expiratory pressure on respiratory resistance in patients with the adult respiratory distress syndrome and in normal anesthetized subjects. Am Rev Respir Dis. 1991;144(1):101–7. 2064114 10.1164/ajrccm/144.1.101

[23] Chiumello D, Carlesso E, Cadringher P, Caironi P, Valenza F, Polli F, et al. Lung stress and strain during mechanical ventilation for acute respiratory distress syndrome. Am J Respir Crit Care Med. 2008;178(4):346–55. 18451319 10.1164/rccm.200710-1589OC

[24] Silva PL, Scharffenberg M, Rocco PRM. Understanding the mechanisms of ventilator-induced lung injury using animal models. Intensive Care Med Experimental. 2023;11(1):82. 10.1186/s40635-023-00569-5 PMC10682329 38010595

[25] Henderson WR, Chen L, Amato MBP, Brochard LJ. Fifty years of research in ARDS. respiratory mechanics in acute respiratory distress syndrome. Am J Respir Crit Care Med. 2017;196(7):822–33. 28306327 10.1164/rccm.201612-2495CI

[26] Chen L, Jonkman A, Pereira SM, Lu C, Brochard L. Driving pressure monitoring during acute respiratory failure in 2020. Curr Opin Crit Care. 2021;27(3):303–10. 33899820 10.1097/MCC.0000000000000827

[27] Chiumello D, Carlesso E, Brioni M, Cressoni M. Airway driving pressure and lung stress in ARDS patients. Crit Care (London England). 2016;20:276. 10.1186/s13054-016-1446-7 PMC4993008 27545828

[28] Amato MB, Meade MO, Slutsky AS, Brochard L, Costa EL, Schoenfeld DA, et al. Driving pressure and survival in the acute respiratory distress syndrome. N Engl J Med. 2015;372(8):747–55. 25693014 10.1056/NEJMsa1410639

[29] Zaidi SF, Shaikh A, Khan DA, Surani S, Ratnani I. Driving pressure in mechanical ventilation: A review. World J Crit Care Med. 2024;13(1):88385. 38633474 10.5492/wjccm.v13.i1.88385 PMC11019631

[30] Haudebourg AF, Tuffet S, Perier F, Razazi K, de Prost N, Mekontso Dessap A et al. Driving pressure-guided ventilation decreases the mechanical power compared to predicted body weight-guided ventilation in the acute respiratory distress syndrome. Critical care (London, England). 2022;26(1):185. 10.1186/s13054-022-04054-5 PMC9208543 35725498

[31] Chiumello D, Brioni M. Severe hypoxemia: which strategy to choose. Crit Care (London England). 2016;20(1):132. 10.1186/s13054-016-1304-7 PMC4891828 27255913

[32] Grasselli G, Calfee CS, Camporota L, Poole D, Amato MBP, Antonelli M, et al. ESICM guidelines on acute respiratory distress syndrome: definition, phenotyping and respiratory support strategies. Intensive Care Med. 2023;49(7):727–59. 37326646 10.1007/s00134-023-07050-7 PMC10354163

[33] Qadir N, Sahetya S, Munshi L, Summers C, Abrams D, Beitler J, et al. An update on management of adult patients with acute respiratory distress syndrome: an official American thoracic society clinical practice guideline. Am J Respir Crit Care Med. 2024;209(1):24–36. 38032683 10.1164/rccm.202311-2011ST PMC10870893

[34] Chiumello D, Dres M, Camporota L. Lung and diaphragm protective ventilation guided by the esophageal pressure. Intensive Care Med. 2022;48(10):1302–4. 35906414 10.1007/s00134-022-06814-x

[35] Jonkman AH, Telias I, Spinelli E, Akoumianaki E, Piquilloud L. The oesophageal balloon for respiratory monitoring in ventilated patients: updated clinical review and practical aspects. Eur Respir Rev. 2023;32(168). 10.1183/16000617.0186-2022 PMC10189643 37197768

[36] Piquilloud L, Beitler JR, Beloncle FM. Monitoring esophageal pressure. Intensive care medicine. 2024. 10.1007/s00134-024-07401-y 38602514

[37] Chiumello D, Caccioppola A, Pozzi T, Lusardi AC, De Giorgis V, Galanti V, et al. The assessment of esophageal pressure using different devices: a validation study. Minerva Anestesiol. 2020;86(10):1047–56. 32538580 10.23736/S0375-9393.20.14458-4

[38] Agostoni E. Mechanics of the pleural space. Physiol Rev. 1972;52(1):57–128. 4550113 10.1152/physrev.1972.52.1.57

[39] Yoshida T, Amato MBP, Grieco DL, Chen L, Lima CAS, Roldan R, et al. Esophageal manometry and regional transpulmonary pressure in lung injury. Am J Respir Crit Care Med. 2018;197(8):1018–26. 29323931 10.1164/rccm.201709-1806OC

[40] Cressoni M, Gotti M, Chiurazzi C, Massari D, Algieri I, Amini M, et al. Mechanical power and development of Ventilator-induced lung injury. Anesthesiology. 2016;124(5):1100–8. 26872367 10.1097/ALN.0000000000001056

[41] Gattinoni L, Tonetti T, Cressoni M, Cadringher P, Herrmann P, Moerer O, et al. Ventilator-related causes of lung injury: the mechanical power. Intensive Care Med. 2016;42(10):1567–75. 27620287 10.1007/s00134-016-4505-2

[42] Chiumello D, Gotti M, Guanziroli M, Formenti P, Umbrello M, Pasticci I, et al. Bedside calculation of mechanical power during volume- and pressure-controlled mechanical ventilation. Crit Care (London England). 2020;24(1):417. 10.1186/s13054-020-03116-w PMC7351639 32653011

[43] Costa ELV, Slutsky AS, Brochard LJ, Brower R, Serpa-Neto A, Cavalcanti AB, et al. Ventilatory variables and mechanical power in patients with acute respiratory distress syndrome. Am J Respir Crit Care Med. 2021;204(3):303–11. 33784486 10.1164/rccm.202009-3467OC

[44] Guerin C, Papazian L, Reignier J, Ayzac L, Loundou A, Forel JM, et al. Effect of driving pressure on mortality in ARDS patients during lung protective mechanical ventilation in two randomized controlled trials. Crit Care. 2016;20(1):384. 27894328 10.1186/s13054-016-1556-2 PMC5126997

[45] Serpa Neto A, Deliberato RO, Johnson AEW, Bos LD, Amorim P, Pereira SM, et al. Mechanical power of ventilation is associated with mortality in critically ill patients: an analysis of patients in two observational cohorts. Intensive Care Med. 2018;44(11):1914–22. 30291378 10.1007/s00134-018-5375-6

[46] Parhar KKS, Zjadewicz K, Soo A, Sutton A, Zjadewicz M, Doig L, et al. Epidemiology, mechanical Power, and 3-Year outcomes in acute respiratory distress syndrome patients using standardized Screening. An observational cohort study. Ann Am Thorac Soc. 2019;16(10):1263–72. 31247145 10.1513/AnnalsATS.201812-910OC PMC6812172

[47] Coppola S, Caccioppola A, Froio S, Formenti P, De Giorgis V, Galanti V, et al. Effect of mechanical power on intensive care mortality in ARDS patients. Crit Care. 2020;24(1):246. 32448389 10.1186/s13054-020-02963-x PMC7245621

[48] Gattarello S, Coppola S, Chiodaroli E, Pozzi T, Camporota L, Saager L, et al. Mechanical power ratio and respiratory treatment escalation in COVID-19 pneumonia: A secondary analysis of a prospectively enrolled cohort. Anesthesiology. 2023;138(3):289–98. 36571571 10.1097/ALN.0000000000004465 PMC9904389

[49] Goligher EC, Costa ELV, Yarnell CJ, Brochard LJ, Stewart TE, Tomlinson G, et al. Effect of Lowering Vt on mortality in acute respiratory distress syndrome varies with respiratory system elastance. Am J Respir Crit Care Med. 2021;203(11):1378–85. 33439781 10.1164/rccm.202009-3536OC

[50] Zalucky AA, Dianti J, Neyton LPA, Sinha P, Liu KD, Matthay MA, et al. Elastance May determine the neuromuscular Blockade effect on mortality in acute respiratory distress syndrome. Am J Respir Crit Care Med. 2025;211(6):966–74. 39998496 10.1164/rccm.202406-1231OC PMC12175940

[51] Piquilloud L. Peep setting: let Us come back to physiology. Curr Opin Crit Care. 2024;30(1):1–3. 38164972 10.1097/MCC.0000000000001129

[52] Gattinoni L, Caironi P, Cressoni M, Chiumello D, Ranieri VM, Quintel M, et al. Lung recruitment in patients with the acute respiratory distress syndrome. N Engl J Med. 2006;354(17):1775–86. 16641394 10.1056/NEJMoa052052

[53] Dellamonica J, Lerolle N, Sargentini C, Beduneau G, Di Marco F, Mercat A, et al. PEEP-induced changes in lung volume in acute respiratory distress syndrome. Two methods to estimate alveolar recruitment. Intensive Care Med. 2011;37(10):1595–604. 21866369 10.1007/s00134-011-2333-y

[54] Del Sorbo L, Tisminetzky M, Chen L, Brochard L, Arellano D, Brito R, et al. Association of lung recruitment and change in recruitment-to-inflation ratio from supine to prone position in acute respiratory distress syndrome. Crit Care (London England). 2023;27(1):140. 10.1186/s13054-023-04428-3 PMC10098997 37055792

[55] Chen D, Heunks L, Pan C, Xie J, Qiu H, Yang Y et al. A Novel Index to Predict the Failure of High-Flow Nasal Cannula in Patients with Acute Hypoxemic Respiratory Failure: A Pilot Study. American journal of respiratory and critical care medicine. 2022. 10.1164/rccm.202203-0561LE PMC9799263 35671485

[56] Chen L, Del Sorbo L, Grieco DL, Junhasavasdikul D, Rittayamai N, Soliman I, et al. Potential for lung recruitment estimated by the recruitment-to-Inflation ratio in acute respiratory distress Syndrome. A clinical trial. Am J Respir Crit Care Med. 2020;201(2):178–87. 31577153 10.1164/rccm.201902-0334OC

[57] Jonson B, Richard JC, Straus C, Mancebo J, Lemaire F, Brochard L. Pressure-volume curves and compliance in acute lung injury: evidence of recruitment above the lower inflection point. Am J Respir Crit Care Med. 1999;159(4 Pt 1):1172–8. 10194162 10.1164/ajrccm.159.4.9801088

[58] Cavalcanti AB, Suzumura EA, Laranjeira LN, Paisani DM, Damiani LP, Guimaraes HP, et al. Effect of lung recruitment and titrated positive End-Expiratory pressure (PEEP) vs low PEEP on mortality in patients with acute respiratory distress syndrome: A randomized clinical trial. JAMA. 2017;318(14):1335–45. 28973363 10.1001/jama.2017.14171 PMC5710484

[59] Mercat A, Richard JC, Vielle B, Jaber S, Osman D, Diehl JL, et al. Positive end-expiratory pressure setting in adults with acute lung injury and acute respiratory distress syndrome: a randomized controlled trial. JAMA. 2008;299(6):646–55. 18270353 10.1001/jama.299.6.646

[60] Suter PM, Fairley B, Isenberg MD. Optimum end-expiratory airway pressure in patients with acute pulmonary failure. N Engl J Med. 1975;292(6):284–9. 234174 10.1056/NEJM197502062920604

[61] Rocha NN, Silva PL, Battaglini D, Rocco PRM. Heart-lung crosstalk in acute respiratory distress syndrome. Front Physiol. 2024;15:1478514. 39493867 10.3389/fphys.2024.1478514 PMC11527665

[62] Battaglini D, Rocco PRM, Editorial. Crosstalk between lung and brain, heart, kidney and vascular system in critical illness. Front Physiol. 2024;15:1516682. 39563937 10.3389/fphys.2024.1516682 PMC11573741

[63] Battaglini D, Rocco PRM. Challenges in transitioning from controlled to assisted ventilation in acute respiratory distress syndrome (ARDS) management. J Clin Med. 2024;13:23. 10.3390/jcm13237333 PMC11642309 39685790

[64] Bianchi I, Grassi A, Pham T, Telias I, Teggia Droghi M, Vieira F, et al. Reliability of plateau pressure during patient-triggered assisted ventilation. Analysis of a multicentre database. J Crit Care. 2022;68:96–103. 34952477 10.1016/j.jcrc.2021.12.002

[65] Grassi A, Bianchi I, Teggia Droghi M, Miori S, Bruno I, Balzani E et al. Increased Driving Pressure During Assisted Ventilation for Hypoxemic Respiratory Failure Is Associated with Lower ICU Survival: The ICEBERG Study. American journal of respiratory and critical care medicine. 2025. 10.1164/rccm.202411-2146OC 40540619

[66] Bellani G, Grassi A, Sosio S, Gatti S, Kavanagh BP, Pesenti A, et al. Driving pressure is associated with outcome during assisted ventilation in acute respiratory distress syndrome. Anesthesiology. 2019;131(3):594–604. 31335543 10.1097/ALN.0000000000002846

[67] Tonelli R, Protti A, Spinelli E, Grieco DL, Yoshida T, Jonkman AH, et al. Assessing inspiratory drive and effort in critically ill patients at the bedside. Crit Care (London England). 2025;29(1):339. 10.1186/s13054-025-05526-0 PMC12315345 40745324

[68] de Vries HJ, Tuinman PR, Jonkman AH, Liu L, Qiu H, Girbes ARJ, et al. Performance of noninvasive airway occlusion maneuvers to assess lung stress and diaphragm effort in mechanically ventilated critically ill patients. Anesthesiology. 2023;138(3):274–88. 36520507 10.1097/ALN.0000000000004467

[69] Pelosi P, Ball L, Barbas CSV, Bellomo R, Burns KEA, Einav S, et al. Personalized mechanical ventilation in acute respiratory distress syndrome. Crit Care (London England). 2021;25(1):250. 10.1186/s13054-021-03686-3 PMC8284184 34271958

[70] Battaglini D, Iavarone IG, Robba C, Ball L, Silva PL, Rocco PRM. Mechanical ventilation in patients with acute respiratory distress syndrome: current status and future perspectives. Expert Rev Med Devices. 2023;20(11):905–17. 37668146 10.1080/17434440.2023.2255521

[71] Alkhalifah AS, Rumindo K, Brincat E, Blanchard F, Helleberg J, Clarke D, et al. Optimizing mechanical ventilation: personalizing mechanical power to reduce ICU mortality - a retrospective cohort study. PLoS ONE. 2025;20(2):e0318018. 39946423 10.1371/journal.pone.0318018 PMC11825045

[72] Chiumello D, Fioccola A. Recent advances in cardiorespiratory monitoring in acute respiratory distress syndrome patients. J Intensive Care. 2024;12(1):17. 38706001 10.1186/s40560-024-00727-1 PMC11070081

[73] Battaglini D, Schultz MJ, Puentes GAC, Marini JJ, Rocco PRM. Imaging and pulmonary function techniques in ARDS diagnosis and management: current insights and challenges. Crit Care (London England). 2025;29(1):282. 10.1186/s13054-025-05520-6 PMC12232599 40619387

[74] Nguyen YL, Angus DC, Boumendil A, Guidet B. The challenge of admitting the very elderly to intensive care. Ann Intensiv Care. 2011;1(1):29. 10.1186/2110-5820-1-29 PMC3224497 21906383

[75] Esteban A, Anzueto A, Frutos-Vivar F, Alía I, Ely EW, Brochard L, et al. Outcome of older patients receiving mechanical ventilation. Intensive Care Med. 2004;30(4):639–46. 14991097 10.1007/s00134-004-2160-5

[76] Papoutsi E, Gkirgkiris K, Tsolaki V, Andrianopoulos I, Pontikis K, Vaporidi K, et al. Association between baseline driving pressure and mortality in very old patients with acute respiratory distress syndrome. Am J Respir Crit Care Med. 2024;210(11):1329–37. 39388641 10.1164/rccm.202401-0049OC

[77] Greener JG, Kandathil SM, Moffat L, Jones DT. A guide to machine learning for biologists. Nat Rev Mol Cell Biol. 2022;23(1):40–55. 34518686 10.1038/s41580-021-00407-0

[78] Rubulotta F, Bahrami S, Marshall DC, Komorowski M. Machine learning tools for acute respiratory distress syndrome detection and prediction. Crit Care Med. 2024;52(11):1768–80. 39133071 10.1097/CCM.0000000000006390

[79] Song D, Chen Q, Huang S, Qiu S, Chen Z, Cai Y, et al. Evaluating the impact of ESICM 2023 guidelines and the new global definition of ARDS on clinical outcomes: insights from MIMIC-IV cohort data. Eur J Med Res. 2025;30(1):51. 39849624 10.1186/s40001-025-02289-w PMC11755903

[80] Pathak A, Marshall C, Davis C, Yang P, Kamaleswaran R. RespBERT: A Multi-Site validation of a natural Language processing Algorithm, of radiology notes to identify acute respiratory distress syndrome (ARDS). IEEE J Biomed Health Inf. 2025;29(2):1455–63. 10.1109/JBHI.2024.3502575 PMC11971015 40030382

[81] Bitker L, Talmor D, Richard JC. Imaging the acute respiratory distress syndrome: past, present and future. Intensive Care Med. 2022;48(8):995–1008. 35833958 10.1007/s00134-022-06809-8 PMC9281340

[82] Chiumello D, Coppola S, Catozzi G, Danzo F, Santus P, Radovanovic D. Lung imaging and artificial intelligence in ARDS. J Clin Med. 2024;13(2). 10.3390/jcm13020305 PMC10816549 38256439

[83] Sathe NA, Xian S, Mabrey FL, Crosslin DR, Mooney SD, Morrell ED, et al. Evaluating construct validity of computable acute respiratory distress syndrome definitions in adults hospitalized with COVID-19: an electronic health records based approach. BMC Pulm Med. 2023;23(1):292. 37559024 10.1186/s12890-023-02560-y PMC10413524

[84] Maddali MV, Churpek M, Pham T, Rezoagli E, Zhuo H, Zhao W, et al. Validation and utility of ARDS subphenotypes identified by machine-learning models using clinical data: an observational, multicohort, retrospective analysis. Lancet Respiratory Med. 2022;10(4):367–77. 10.1016/S2213-2600(21)00461-6 PMC8976729 35026177

[85] Baedorf-Kassis EN, Glowala J, Póka KB, Wadehn F, Meyer J, Talmor D. Reverse triggering neural network and rules-based automated detection in acute respiratory distress syndrome. J Crit Care. 2023;75:154256. 36701820 10.1016/j.jcrc.2023.154256 PMC10173144

[86] Stivi T, Padawer D, Dirini N, Nachshon A, Batzofin BM, Ledot S. Using artificial intelligence to predict mechanical ventilation weaning success in patients with respiratory Failure, including those with acute respiratory distress syndrome. J Clin Med. 2024;13(5). 10.3390/jcm13051505 PMC10934889 38592696

[87] Hartman E, Scott AM, Karlsson C, Mohanty T, Vaara ST, Linder A, et al. Interpreting biologically informed neural networks for enhanced proteomic biomarker discovery and pathway analysis. Nat Commun. 2023;14(1):5359. 37660105 10.1038/s41467-023-41146-4 PMC10475049

[88] Pennati F, Aliverti A, Pozzi T, Gattarello S, Lombardo F, Coppola S, et al. Machine learning predicts lung recruitment in acute respiratory distress syndrome using single lung CT scan. Ann Intensiv Care. 2023;13(1):60. 10.1186/s13613-023-01154-5 PMC10322807 37405546

